# Positive Effects of Nutritional Education in a Patient With Pleuroparenchymal Fibroelastosis

**DOI:** 10.7759/cureus.78976

**Published:** 2025-02-13

**Authors:** Hideaki Yamakawa, Hiroki Ohta, Shintaro Sato, Hidekazu Matsushima

**Affiliations:** 1 Respiratory Medicine, Saitama Red Cross Hospital, Saitama, JPN

**Keywords:** body weight, dietitian nutritionists, interstitial lung disease, nutrition therapy, pleuroparenchymal fibroelastosis

## Abstract

Pleuroparenchymal fibroelastosis (PPFE) patients often experience dyspnea, which can lead to weight loss due to increased calorie expenditure from the effort required for breathing. Weight loss in PPFE has been associated with a decline in lung function, which, in turn, may contribute to a poor prognosis. Therefore, nutritional therapy plays a critical role in managing this condition. Here, we report a case of a patient with idiopathic PPFE (iPPFE) who showed a clear positive response to nutritional therapy. The patient experienced a gradual decline in lung function (FVC) and body weight, leading to worsening respiratory symptoms. After being referred to our hospital, a chest CT confirmed the diagnosis of PPFE, and he began nutritional education focused on a high-fat, high-protein diet. Despite initial weight loss, his weight and FVC improved, and his respiratory symptoms eased. No pharmacological intervention was implemented throughout the course. The patient reported positive effects from nutritional therapy and remains under follow-up care. Our findings suggest that maintaining body weight and physique may help stabilize lung function and potentially slow the progression of PPFE.

## Introduction

Pleuroparenchymal fibroelastosis (PPFE) is a rare lung disease first identified by Amitani et al. as idiopathic upper-lobe fibrosis, characterized by pleural fibrosis and subjacent parenchymal fibroelastosis predominantly affecting the upper lobes [1,2]. Idiopathic PPFE (iPPFE) was classified as a rare idiopathic interstitial pneumonia (IIP) in the 2013 international classification update [3]. In Japan, a diagnostic criterion has been proposed, defining PPFE as a condition characterized by an insidious onset of dry cough or exertional dyspnea, multiple subpleural foci of airspace consolidation with traction bronchiectasis in the bilateral upper lobes on high-resolution computed tomography (HRCT), and subpleural zonal or wedge-shaped dense fibrosis with alveolar collapse, collagen deposition, and septal elastosis, with or without visceral pleural thickening [4]. This comprehensive approach facilitates accurate diagnosis in clinical practice.

Although the prevalence and incidence of iPPFE remain unknown, it has been reported to account for approximately 0.5-10.4% of IIPs [5]. Unlike chronic obstructive pulmonary disease (COPD), PPFE patients are generally more likely to be non-smokers, with reported rates ranging from 30.6% to 75.0% [5]. The exact cause of PPFE remains unclear; however, it has been associated with drug exposure, chronic hypersensitivity pneumonia, chronic infections, collagen vascular diseases, and bone marrow transplantation [2].

Currently, no pharmacological treatment for iPPFE has been established, and lung transplantation remains the only curative option for advanced cases [2,5]. PPFE patients often experience dyspnea, leading to significant weight loss due to excessive caloric expenditure from the increased effort required to breathe [2,3]. Therefore, nutritional therapy and rehabilitation are considered crucial, although no definitive evidence supporting their effectiveness has been reported to date.

Here, we present a case in which nutritional therapy was introduced for a patient experiencing weight loss, resulting in the maintenance of body weight and physique, as well as the stabilization of lung function.

## Case presentation

**Figure 1:**
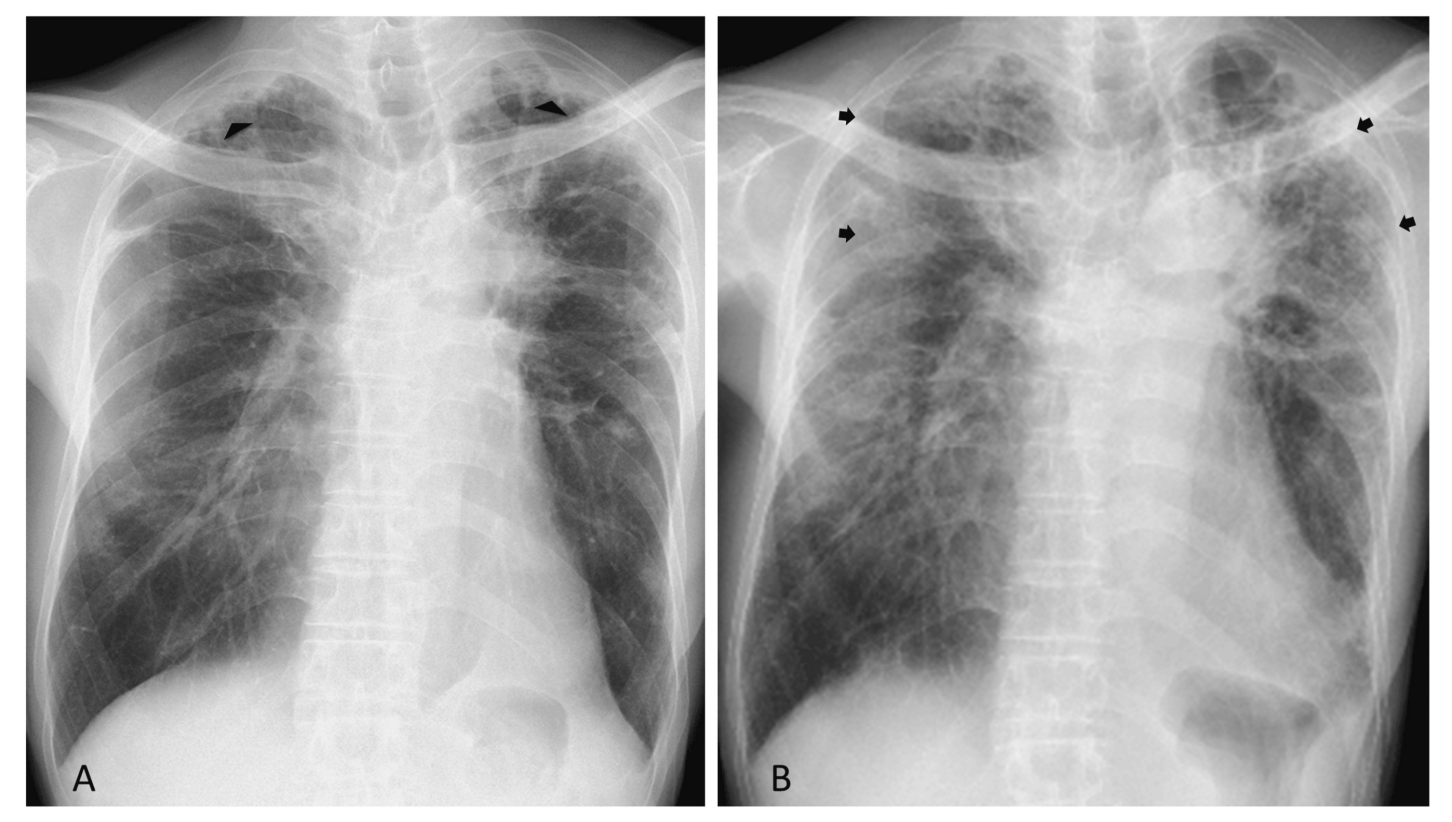
Chest X-ray findings. (A) In May 2018, a chest X-ray revealed irregular apical thickening (arrow heads) and reticular opacities in both upper lung fields. (B) In June 2023, a chest X-ray showed progression of fibrotic changes, with increasing reticular shadows and ground-glass opacities (arrows) in the upper lung fields, accompanied by an upward displacement of the hilar structures and a reduction in the volume of the upper lung lobes.

In May 2018, the patient was diagnosed with iPPFE at another hospital following the detection of abnormal chest shadows (Figure 1A). He had a smoking history of 20 cigarettes per day for 20 years but had no obvious history of dust inhalation. Initially asymptomatic, he was under observation, with a forced vital capacity (FVC) of 73.3% in May 2018. However, it gradually declined to 58.3% in November 2020 and 52.9% in August 2022, accompanied by the onset of exertional dyspnea and radiological progression, characterized by an upward shift of hilar structures with the reduction of upper lung lobe volume (Figure 1B).

**Figure 2:**
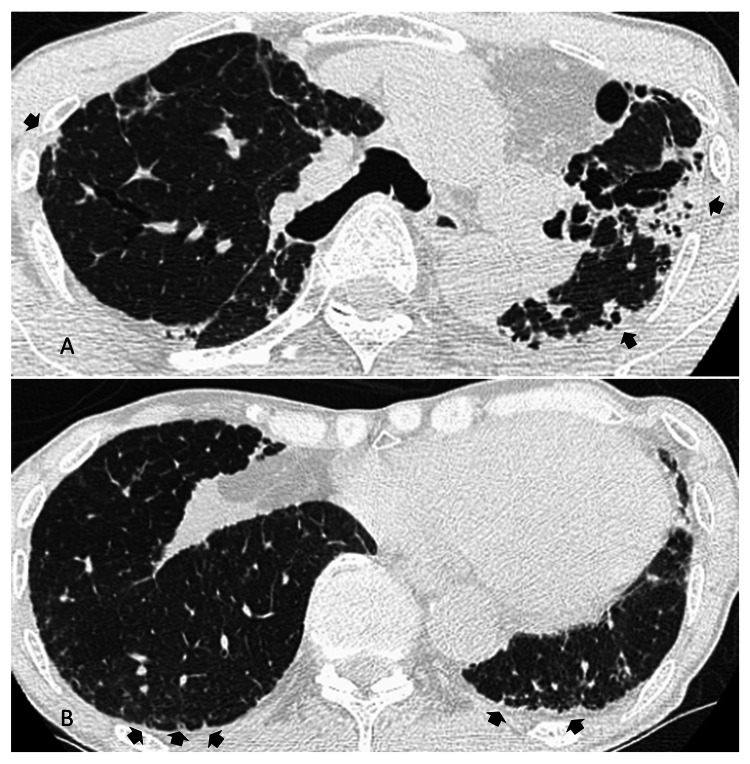
Chest computed tomography images. (A) In the upper-lung zone, parenchymal fibrotic opacities with traction bronchiectasis (arrows) were shown. (B) In the lower-lung zone, lesions are minimal, with only mild reticular changes (arrows) observed at the base of both the lungs.

His body weight also decreased from 54.3 kg in May 2018 to 52.6 kg in November 2020 and 51.0 kg in August 2022. Regarding the weight loss, the possibility of malignancy was considered; however, upper endoscopy and whole-body CT findings were negative. In June 2023, at the age of 74, he was referred to our hospital. A chest CT scan showed fibrotic opacities in the subpleural lung parenchyma with traction bronchiectasis in the bilateral upper lobes, which are consistent with PPFE (Figure 2A, 2B).

**Figure 3:**
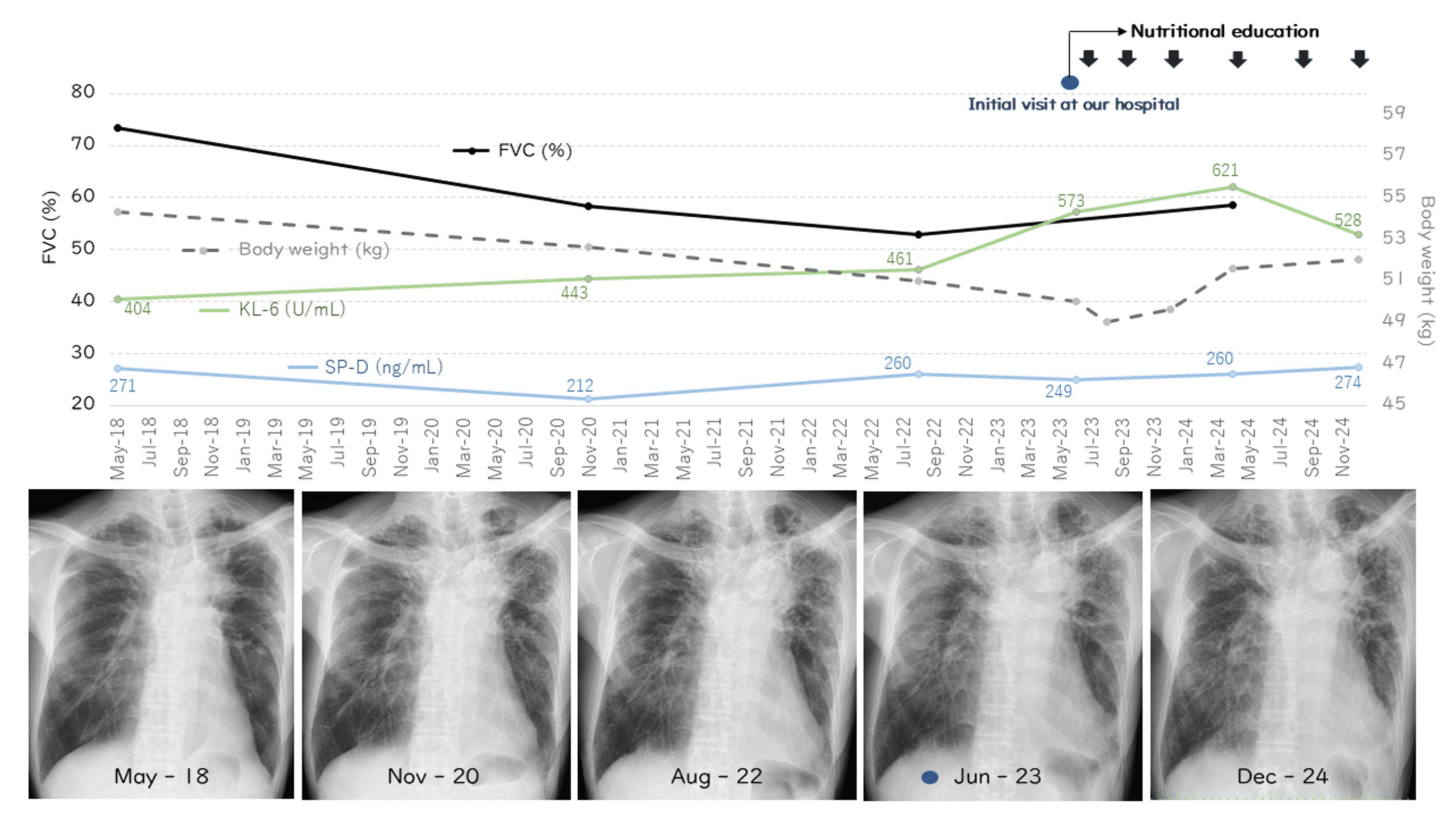
Physiological and radiological course. His forced vital capacity (FVC) declined from 73.3% in May 2018 to 52.9% in August 2022, accompanied by worsening dyspnea and weight loss. After starting nutritional education in July 2023, his weight, which had dropped to 49 kg in August 2023, increased to 51.6 kg by April 2024. FVC improved to 58.5%, and by December, his weight had reached 52.0 kg, with reduced symptoms. From 2018 to 2023, chest X-rays showed progressive fibrosis with increasing reticular shadows and upper lobe volume loss. However, after starting nutritional therapy, no obvious radiological progression was observed through December 2024. The interstitial lung disease (ILD) marker KL-6 showed a slight increasing trend from the initial visit to our hospital in June 2023 until April 2024, after which it began to decline. Surfactant protein D (SP-D) remained relatively stable with only mild fluctuations.

Arterial blood gas analysis on room air showed a pH of 7.4, a PO₂ of 86.6 Torr, and a PaCO₂ of 46.1 Torr, indicating a mild tendency toward CO₂ retention. The ILD markers KL-6 and SP-D were mildly elevated at 573 U/mL and 249 ng/mL, respectively. At the initial visit to our hospital, the patient’s body weight was 50.0 kg. Nutritional education by dietitian nutritionists began in July at a frequency of once every two to four months. The dietitian recommended a high-fat, high-protein diet with moderate carbohydrates, based on nutritional therapy for patients with COPD [6], with a total calorie goal set at 2200 kcal/day, to ensure that the patient does not lose any more weight at the very least. Although the patient’s body weight decreased to 49.0 kg in August, it rebounded to 49.6 kg in December and further improved to 51.6 kg by April 2024. In addition, lung function showed improvement, with FVC increasing to 58.5%. Additional information includes residual volume/total lung capacity (RV/TLC) at 42.3%, functional residual capacity (FRC) at 47.8%, and diffusing capacity of the lung for carbon monoxide (DLCO) at 80.3%. By December, his body weight had increased to 52.0 kg, and his respiratory symptoms had eased, and no obvious radiological progression was observed (Figure 3).

The patient himself reported feeling the positive effects of the nutritional education. Throughout the course, no pharmacological intervention was administered. Currently, he remains under follow-up care in our outpatient clinic.

## Discussion

PPFE has recently been recognized as a distinct clinicopathological condition [1,2]. The most common symptoms at presentation include exertional dyspnea, cough, weight loss, pneumothorax, and chest pain [1,2,4,5,7]. This case represents a valuable report in which progressive weight loss and worsening imaging findings were observed but were successfully addressed following the initiation of nutritional counselling by a dietitian. The intervention not only halted weight loss but also led to weight gain, accompanied by improvements in respiratory symptoms and lung function.

Weight loss is common in PPFE and has been reported to correlate with the progression of lung function decline [1,2,4,5,7,8]. Similarly, low body weight and weight loss are poor prognostic factors in any lung disease [9-11]. While weight loss is often considered a consequence of disease progression and severity, as seen in this case, maintaining or improving body weight and physique may play a protective role against the worsening of lung lesions. In COPD, weight gain has been reported to improve lung function by maintaining body composition and preserving respiratory muscle function [6]. Therefore, nutritional therapy is a critical treatment approach that should be considered alongside pharmacological interventions. This case highlights that the maintenance of body weight and physique through nutritional support can be a crucial therapy for improving prognosis. Moreover, careful monitoring of body weight changes may provide valuable information for predicting outcomes in patients with PPFE. The ideal approach involves early intervention, as early nutritional education and treatment can help maintain body weight and potentially prolong survival. Clinicians should recognize the importance of initiating nutritional interventions at an earlier stage, even when the disease appears to be mild.

## Conclusions

We present a case of a PPFE patient who demonstrated a clear positive response to nutritional therapy. In PPFE, low body weight and weight loss are associated with a worse prognosis. In cases of severe interstitial lung disease, increased calorie expenditure leads to weight loss. Conversely, as body weight and physical strength decline, respiratory muscles weaken, exacerbating restrictive ventilatory impairment. This represents a "chicken and egg" relationship. However, in this case, despite the absence of pharmacological intervention, nutritional therapy successfully maintained body weight while also preserving lung function. This suggests that nutritional therapy may be a viable treatment option. To establish specific nutritional intake targets for individual patients, further studies with a larger number of cases are needed. As clinicians, we must recognize the importance of nutritional therapy in addition to pharmacological treatment.
